# Supplementary material for: A chlorophyll halo over Maud Rise in the Southern Ocean
Source: Nat Commun. 2025 Dec 2;16:11302. doi: 10.1038/s41467-025-66458-5 (PMC12722387; doi:10.1038/s41467-025-66458-5)
Supplement: Supplementary file 1 — Supplementary Information [file 41467_2025_66458_MOESM1_ESM.pdf]

A Chlorophyll Halo over Maud Rise in the Southern Ocean

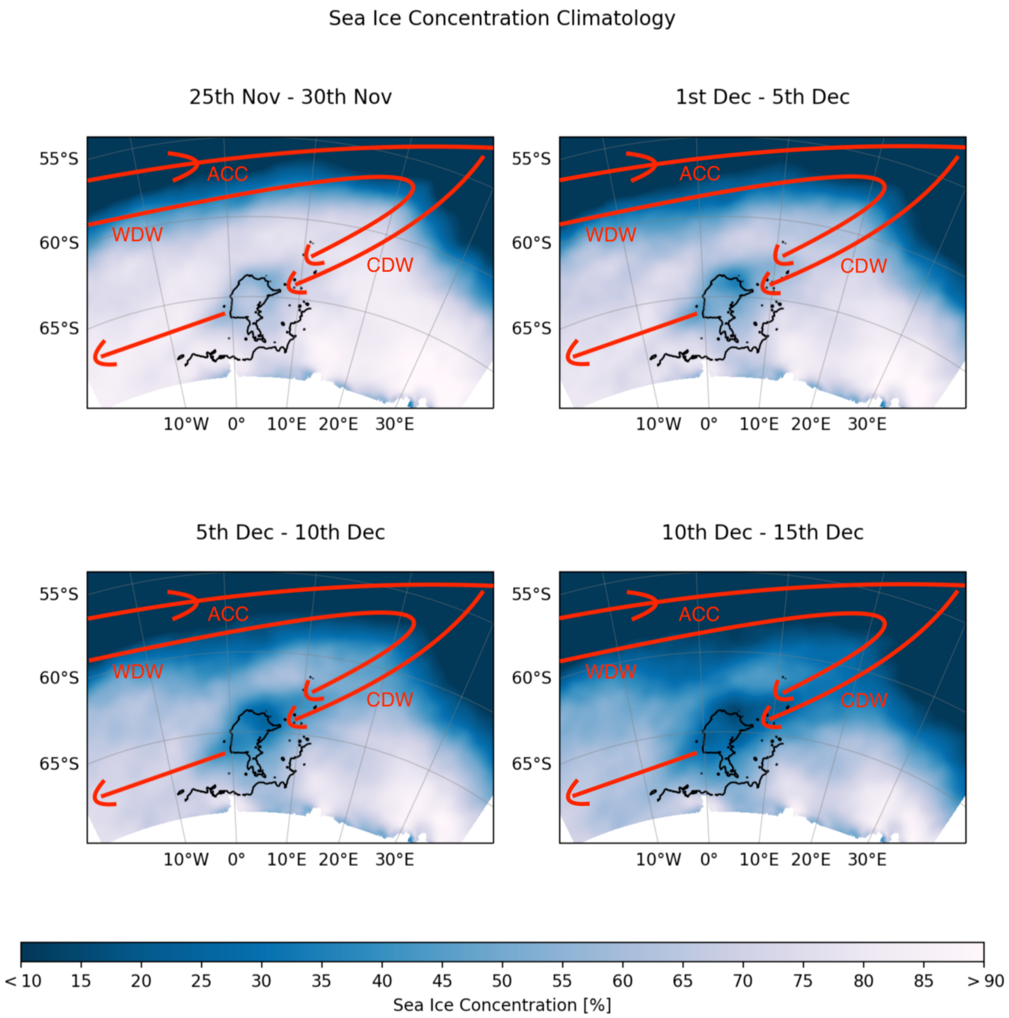

Figure S1: **Sea Ice Concentration Climatology of the Austral Summer :** spatial maps of sea ice concentration [%] obtained by averaging satellite-derived data over 4 different periods from 1998 to 2022. The 3800 meter isobath is drawn as a solid black line. Major currents in the Weddell Gyre are indicated. ACC, WDW and CDW stand for Antarctic Circumpolar Current, Warm Deep Water and Cold Deep Water respectively.

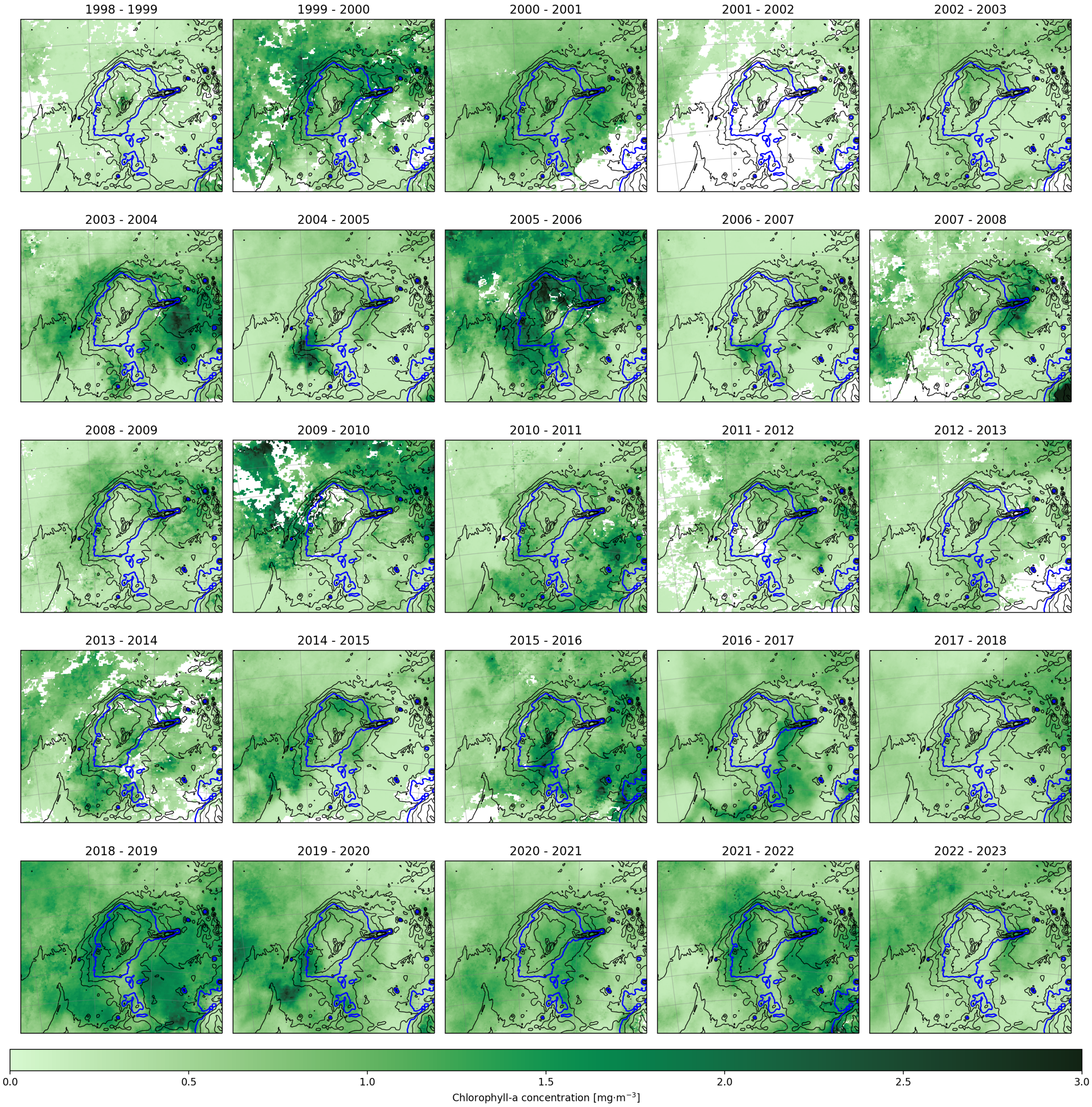

Figure S2: **Annual December climatologies of the chlorophyll halo.** Spatial maps of Chl-a concentration obtained by averaging satellite data over December for each year. Pixels with no data are drawn in white. Isobaths, plotted as black solid contours, are drawn every 500 meters. The 3500 meter isobath is drawn in blue.

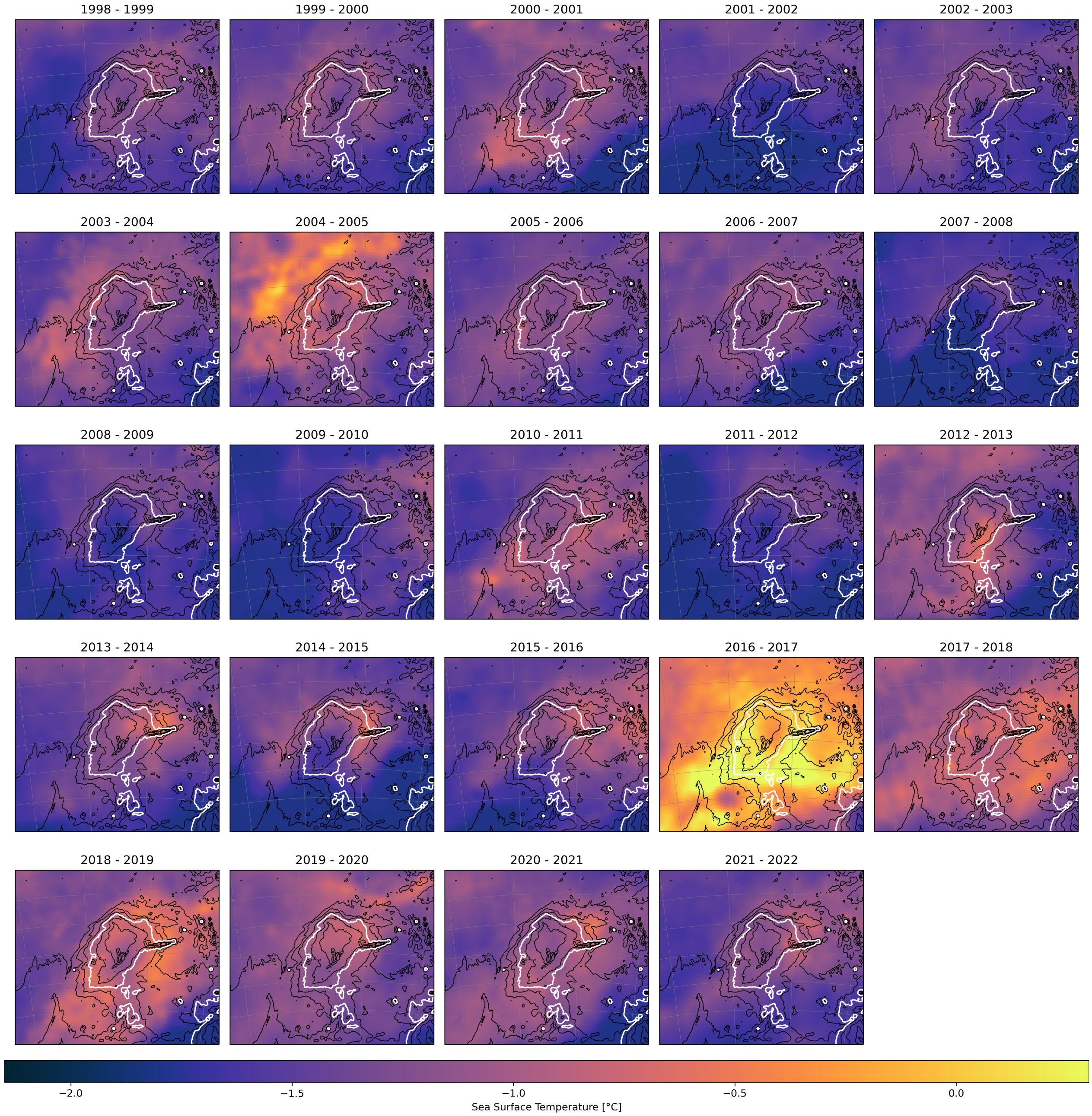

Figure S3: **Annual December climatologies of the warm water halo.** Spatial maps of sea surface temperature obtained by averaging satellite data over December for each year. Isobaths, plotted as black solid contours, are drawn every 500 meters. The 3500 meter isobath is drawn in white.

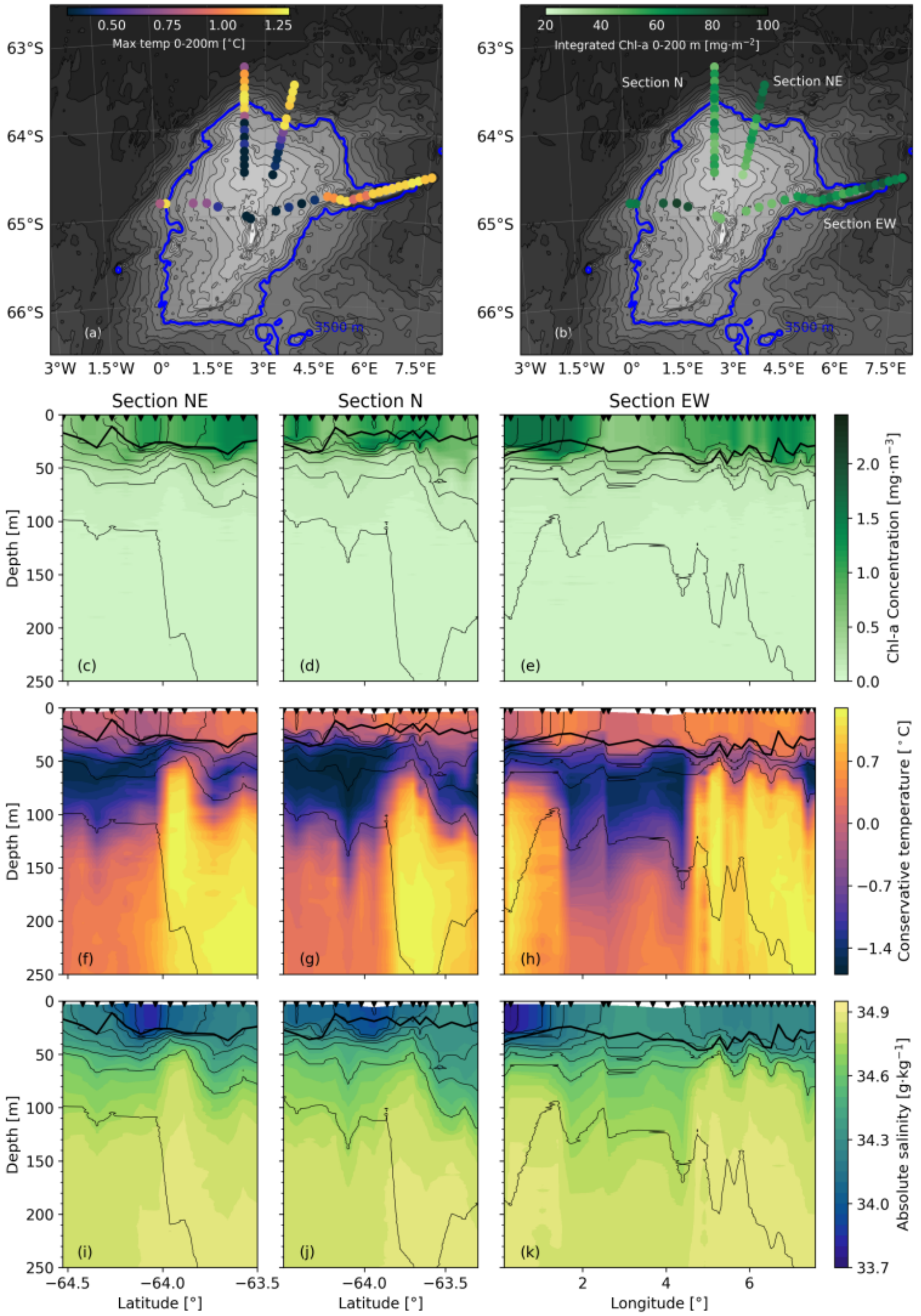

Figure S4: **CTD measurements over Maud Rise of Chlorophyll-*a*, Sea Water Temperature and Salinity.** (a) Maximum temperature measured between 0 and 200 meters at each CTD cast. (b) Integrated Chl-*a* concentration between 0 and 200 meters. For both (a) and (b), isobaths are drawn every 200 meters. The 3500 meter isobath is shown in blue. CTD sections of (c-e) Chl-*a*, (f-h) temperature, and (i-k) salinity for Section NE, Section N and Section EW, as marked in b). Isopycnals are drawn as thin black lines every 0.1 mg·m^{-3}. The thick black line in (c-k) is the mixed layer depth based on the definition used in Xing et al. [46].

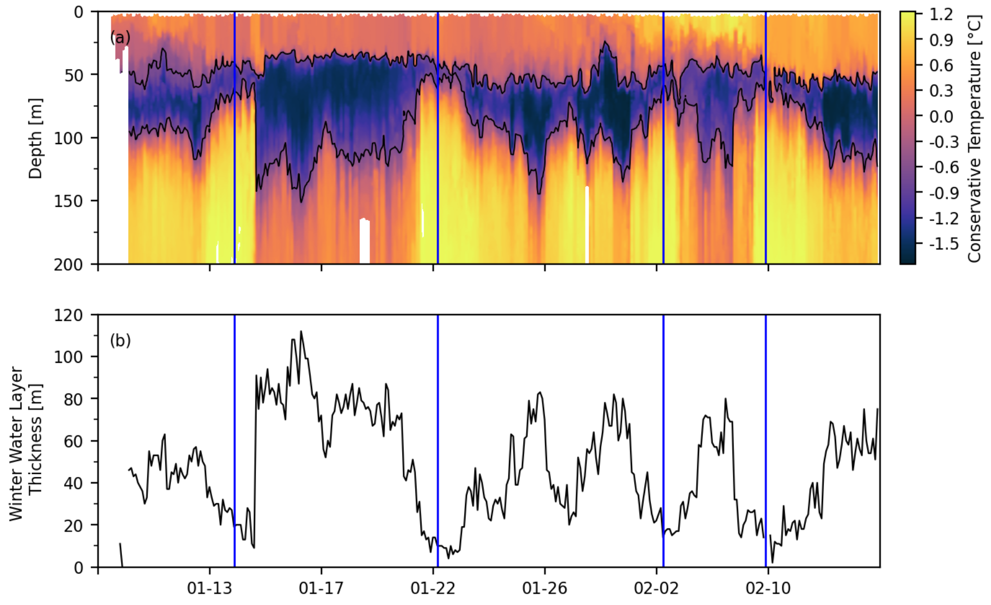

Figure S5: **Evolution of the cold Winter Water thickness.** a) conservative temperature measured by SG640. The two dashed lines represent the -0.7 °C isotherms that define the Cold Water Layer. b) Thickness computed from the depth differences of the two aforementioned isotherms. The two pairs of blue lines in both panels mark the first four crossings.

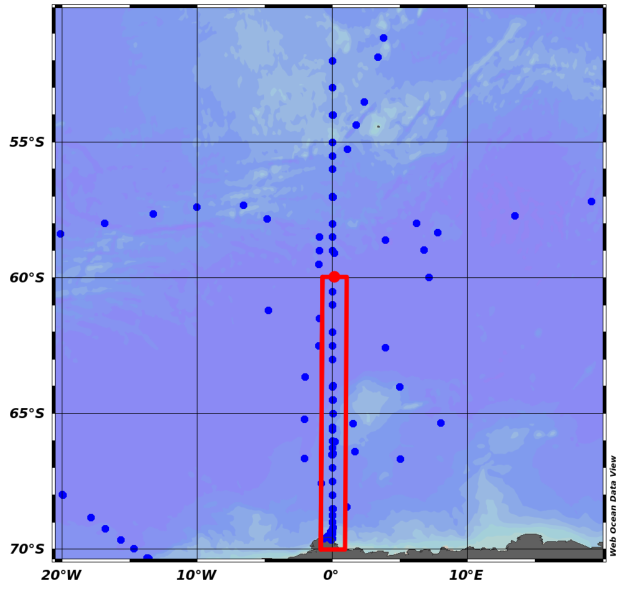

Figure S6: **All stations with δ^3He measurements.** The boundaries of the region are (20°W-20°E,50°S-70°S). The red box encircled stations whom datasets are used for plotting the transects of Figure S7

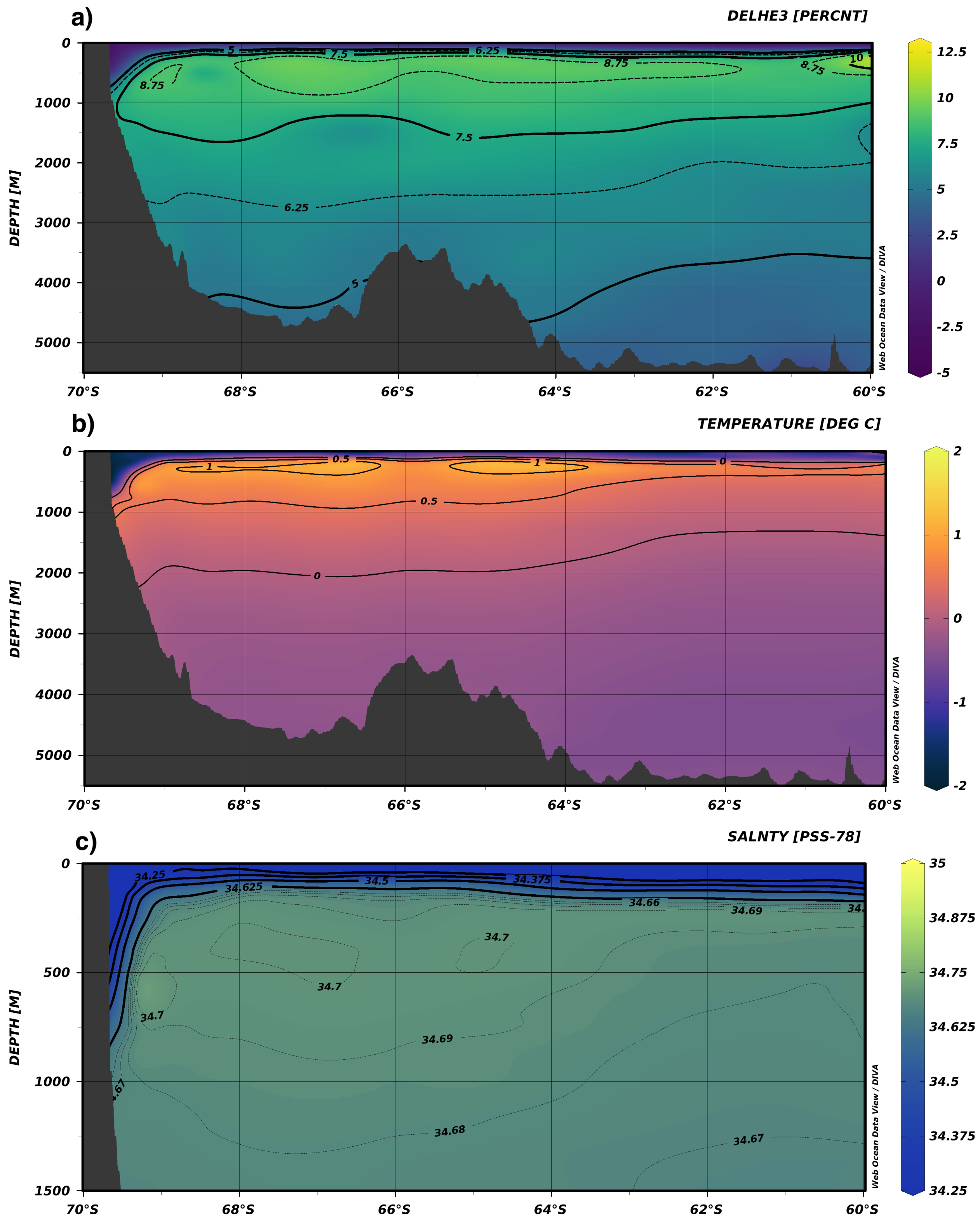

Figure S7: δ^3**He measurements and water masses identification.** Transects along the 0° meridian from 60°S to 70°S of a) δ^3He b) temperature, c) salinity. Isolines are drawn in black.

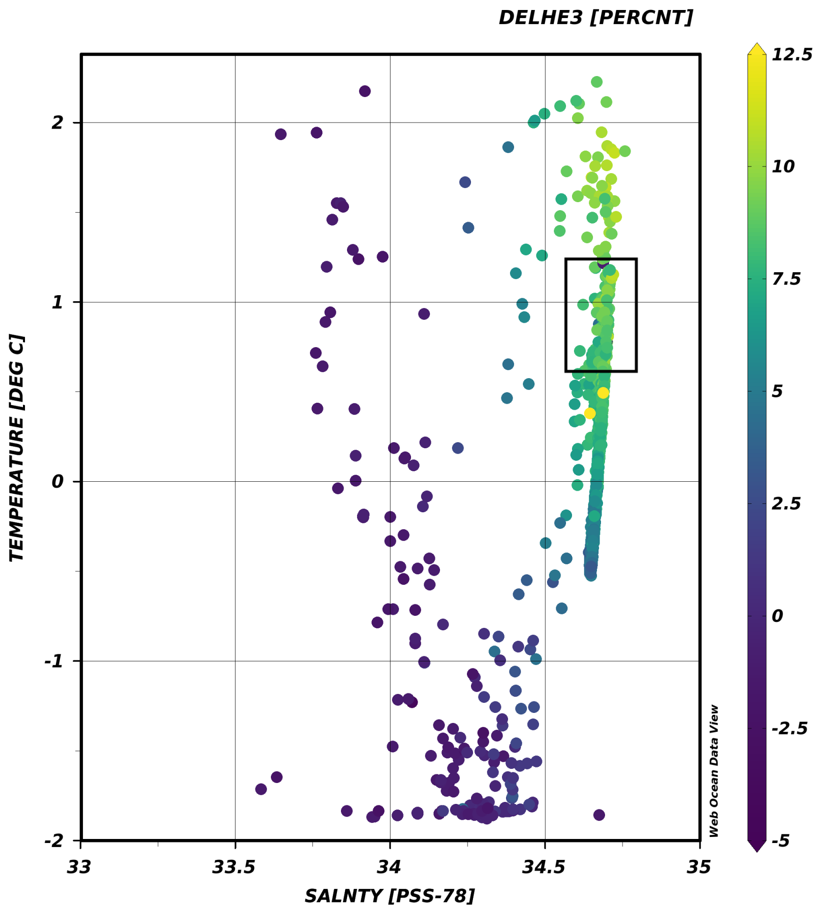

Figure S8: **Temperature/Salinity diagram.** All δ^3He measurements of the GLODAP database in the region (20°W-20°E,50°S-70°S) are represented as dots with color varies according to the corresponding δ^3He value. Measurements of water masses with a salinity of ≈ 34.69 PSS-78 and a temperature of ≈ 1°C are highlighted by a black rectangle.

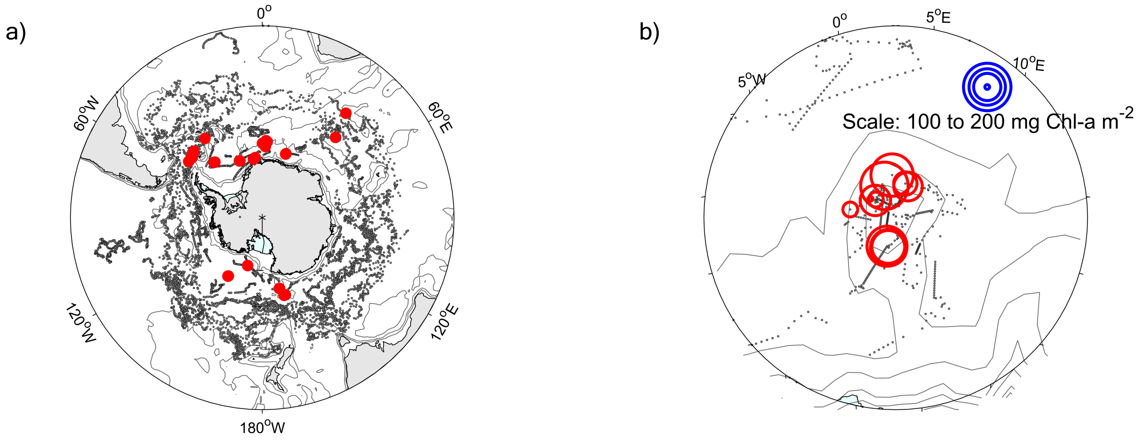

Figure S9: **Relevance of Maud Rise to the Southern Ocean ecosystem.** a) Southern Ocean map with all BGC-Argo floats profile locations available for the period 2012-2020 (dark grey dots) with the profiles of integrated Chl-a > 100 mg·m^{-2} highlighted as red dots. Grey contours are the bathymetry and the grey areas show land. a) Map with all the BGC-Argo floats profiles with integrated Chl-a over 100 mg·m^{-2} (red dots). Grey dots represent all BGC-Argo float profiles for the period 2012-2020, i.e., 8560 profiles of Chl-a fluorescence. Chl-a fluorescence is corrected with the Roesler et al. algorithm [50], and corrected for Non-Photochemical Quenching, [46]. b) similar to a) around Maud Rise and with red dots size scaled by the integrated Chl-a biomass over the upper 200 meters depth. For scaling, blue circles are indicating integrated Chl-a values of, from smallest to largest, 100, 125, 150 and 200 mg·m^{-2}

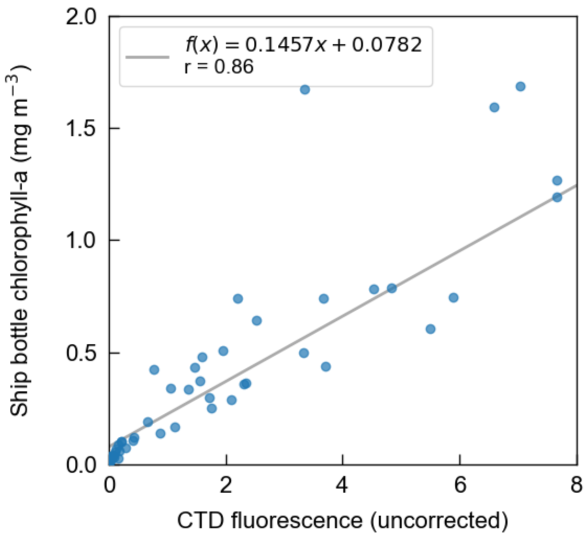

Figure S10: **CTD calibration against in-situ bottle samples.**

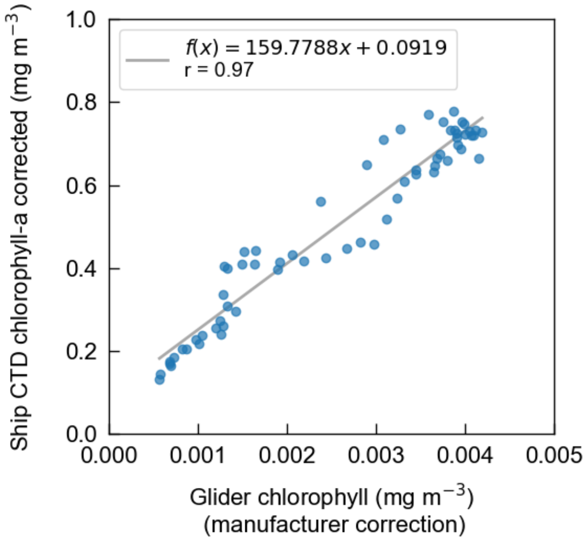

Figure S11: **SG640 Chlorophyll-a (mg·m^{-3}) calibration against calibration CTD cast.**

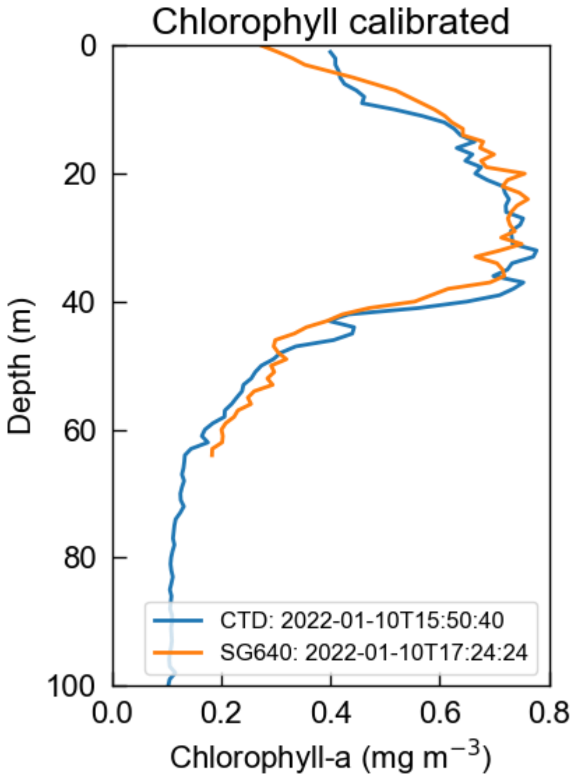

Figure S12: **SG640 Chlorophyll-a (mg·m^{-3}) calibration profile comparison to the Chlorophyll-a (mg·m^{-3}) calibration profile of the CTD cast.**

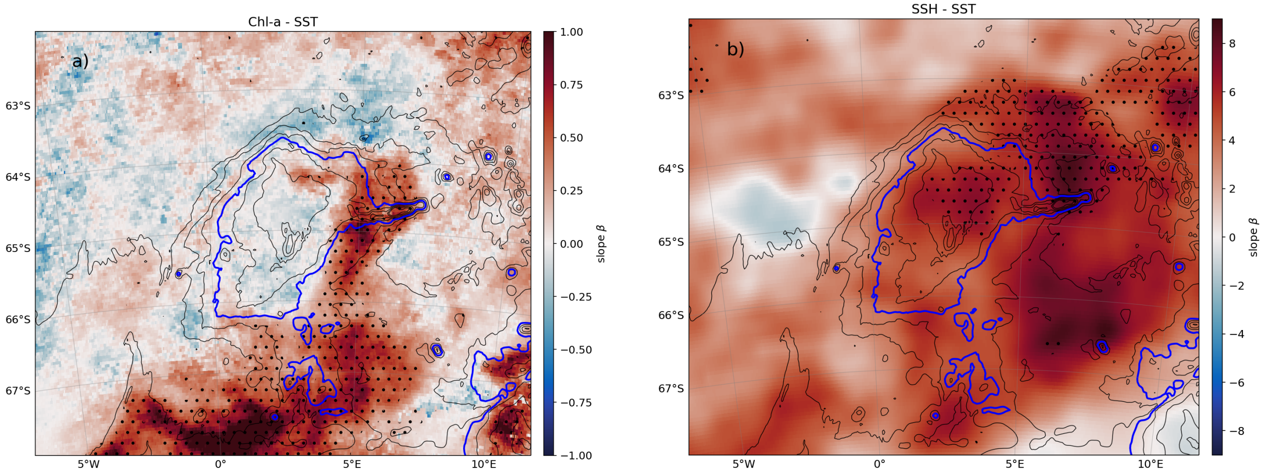

Figure S13: **Slope of the correlation.** a) Slope of the Chl-a -SST correlation b) Slope of the SSH-SST correlation. Isobaths, plotted as black solid contours, are drawn every 500 meters. The 3500 meter isobath is drawn in Blue. Hatched areas show significant correlations ($p < 0.05$).
